# Prospective evaluation of multitarget treatment of pediatric patients with helical intensity-modulated radiotherapy

**DOI:** 10.1007/s00066-020-01670-4

**Published:** 2020-08-03

**Authors:** Maria-Elena A. Salfelder, Kerstin A. Kessel, Uwe Thiel, Stefan Burdach, Severin Kampfer, Stephanie E. Combs

**Affiliations:** 1 Department of Radiation Oncology, Technical University Munich (TUM), Ismaninger Straße 22, 81675 Munich, Germany; 2 DKTK Partner Site Munich, Deutsches Konsortium für Translationale Krebsforschung (DKTK), Munich, Germany; 3 Institute of Radiation Medicine (IRM), Helmholtz Zentrum München, Ingolstädter Landstraße 1, Neuherberg, Germany; 4 Department of Pediatrics and Children’s Cancer Research Center, Kinderklinik München Schwabing, Technical University of Munich School for Medicine, Munich, Germany

**Keywords:** Tomotherapy, Pediatric sarcoma, Toxicity, Survival, Multitarget radiation therapy

## Abstract

**Background and purpose:**

Radiotherapy (RT) is persistently gaining significance in the treatment of pediatric tumors. However, individual features of a growing body and multifocal stages complicate this approach. Tomotherapy offers advantages in the treatment of anatomically complex tumors with low risks of side effects. Here we report on toxicity incidence and outcome of tomotherapy with a focus on multitarget RT (mtRT).

**Materials and methods:**

From 2008 to 2017, 38 children diagnosed with sarcoma were treated with tomotherapy. The median age was 15 years (6–19 years). Toxicity was graded according to the Common Terminology Criteria for Adverse Events v.4.03 and classified into symptoms during RT, acutely (0–6 months) and late (>6 months) after RT, and long-term sideeffects (>24 months).

**Results:**

The main histologies were Ewing sarcoma (*n* = 23 [61%]) and alveolar rhabdomyosarcoma (*n* = 5 [13%]). RT was performed with a median total dose of 54 Gy (40.5–66.0 Gy) and a single dose of 2 Gy (1.80–2.27 Gy). Twenty patients (53%) received mtRT. Median follow-up was 29.7 months (95% confidence interval 15.3–48.2 months) with a 5-year survival of 55.2% (±9.5%). The 5‑year survival rate of patients with mtRT (*n* = 20) was 37.1 ± 13.2%, while patients who received single-target RT (*n* = 18) had a 5-year survival rate of 75 ± 10.8%. Severe toxicities (grade 3 and 4) emerged in 14 patients (70%) with mtRT and 7 patients (39%) with single-target RT. Two non-hematological grade 4 toxicities occurred during RT: one mucositis and one radiodermatitis. After mtRT 5 patients had grade 3 toxicities acute and after single-target RT 4 patients. One patient had acute non-hematological grade 4 toxicities (gastritis, pericarditis, and pericardial effusion) after mtRT. Severe late effects of RT occurred in 2 patients after mtRT and in none of the single-target RT patients. No severe long-term side effects appeared.

**Conclusion:**

Our results showed acceptable levels of acute and late toxicities, considering the highly advanced diseases and multimodal treatment. Hence, tomotherapy is a feasible treatment method for young patients with anatomically complex tumors or multiple targets. Especially mtRT is a promising and innovative treatment approach for pediatric sarcomas, delivering unexpectedly high survival rates for patients with multifocal Ewing sarcomas in this study, whereby the limited number of patients should invariably be considered in the interpretation.

**Electronic supplementary material:**

The online version of this article (10.1007/s00066-020-01670-4) contains supplementary material, which is available to authorized users.

## Introduction

Sarcomas are a heterogeneous group of malignant mesenchymal tumors that make up less than 1% of all human cancers, but up to 20% of pediatric tumors [1]. They are characterized by highly aggressive malignancy and high rates of metastatic spread and recurrences. Although localized sarcomas can commonly be cured using chemotherapy in combination with surgery- and radiotherapy-based local therapy, metastatic spread is often present at diagnosis [1, 2].

Radiotherapy (RT) is persistently gaining significance in the treatment of pediatric tumors [3]. Individual features of a growing body and multifocal stages of pediatric malignancies, especially of sarcomas, complicate this therapeutic approach. Furthermore, new treatment concepts for multifocal stages of pediatric sarcomas include the application of high-dose chemotherapy, such as busulfan and melphalan, which are suspected to highly interact with RT and cause severe side effects but also improve the diagnosis and outcome of Ewing sarcoma [4, 5]. Additionally, the risks of long-term toxicities of RT in childhood cancers have not been adequately assessed due to limited data. These long-term side effects include endocrine dysfunctions, growth delay, and development disorders [6].

Nevertheless, there is a higher risk of developing second malignancies compared to adult patients, which may lead to a decrease in overall survival and quality of life after treatment [7–9]. In order to reduce side effects and long-term toxicity, intensity-modulated radiation therapy (IMRT) may constitute an adequate therapeutic approach. IMRT allows precise irradiation of tumor sites by accumulating the irradiation dose in the defined target volume and minimizing dose to nearby organs at risk [10].

Furthermore, image-guided radiation therapy (IGRT) increases the accuracy of RT based on daily imaging of the tumor sites and adjusting the patient’s position. Thus, IGRT adapts to the position of the patient by comparing images of, e.g., computed tomography (CT), with the original images of the planning CT before each fraction of RT [11].

Helical intensity-modulated RT (tomotherapy) with integrated image guidance can be used to hit multiple targets in one session and offers treatment with a low risk of side effects by reducing the exposed healthy tissue. A further advantage of tomotherapy is its helical delivery pattern of irradiation. While the patient is continuously translated into the gantry, the fan beam rotates continuously around the patient, allowing irradiation of large and anatomically complex tumor volumes [12].

RT enhances not only the local control of tumors but also improves the outcome of high-risk patients with radiosensitive Ewing sarcoma and rhabdomyosarcoma [13, 14]. However, RT also influences the outcome of patients with radioresistant osteosarcomas, where the surgical local control of lesions is difficult or as a treatment method for palliative pain relief [15, 16]. Still, the clinical outcomes and toxicity incidence of pediatric sarcomas treated with RT, especially regarding long-term side effects, remain unclear. This study aims to report on acute and long-term toxicity rates and the outcome of tomotherapy treatment in pediatric sarcomas. A particular focus lies on survival data and toxicity after multitarget RT (mtRT) of children with multifocal tumor stages.

## Patients and methods

### Patients

Between 2008 and 2017, a total of 38 children diagnosed with sarcoma were treated with tomotherapy at our department. In this retrospective evaluation, children with age up to 19 years and a diagnosis of sarcoma were included. A different entity, an age older than 19 years, and irradiation other than tomotherapy led to an exclusion. Table 1 displays the prevalent histological types of sarcomas. The median age was 15 years (range 6–19 years). The local ethics committee of the Medical Faculty of the Technical University München (TUM) approved the study (vote numbers: 470/17 S).

**Table 1:** The prevalent types of sarcomas

Histological types of sarcomas	*n* (%)
*Ewing’s sarcoma*	23 (61%)
*Rhabdomyosarcoma*	6 (16%)
Embryonal Rhabdomyosarcoma	1 (3%)
Alveolar Rhabdomyosarcoma	5 (13%)
*Osteosarcoma*	2 (5%)
*Nonrhabdomyosarcoma soft tissue Sarcoma*	7 (18%)
Synovial sarcoma	2 (5%)
Desmoid sarcoma	2 (5%)
Unclassified sarcoma	2 (5%)
Fibromyxoid sarcoma	1 (3%)

### Treatment characteristics

All patients were treated with helical intensity-modulated radiotherapy (Tomotherapy Hi-ART, Accuray, Sunnyvale, CA, USA). Tomotherapy combines highly precise rotational dose delivery with megavoltage computed tomography (MVCT). The rotating gantry system allows the best target coverage for multitarget, complex, and very long volumes, which can be targeted from any angle to deliver a highly conformal dose distribution to the target while simultaneously sparing healthy tissue [17]. For treatment planning, a contrast-enhanced planning CT was generally used to define organs at risk and the gross tumor volumes. Target volume definition was performed with the goal of small treatment regions to avoid normal tissue toxicity, especially in patients with higher numbers of targets. Therefore, the concept was based on Euro-Ewing, but with much smaller safety margins of approximately 5 mm. In detail, the gross tumor volume (GTV) with an additional margin of approximately 5 mm resulted in the clinical target volume (CTV). A planning target volume (PTV) was added based on the institutional standard. Immobilization during RT was ensured by using a blue bag vacuum mattress, wingboard, and masks when necessary, depending on the locations treated. All patients were treated with a frequency of five fractions per week, with a median single dose of 2 Gy (range 1.8–2.27 Gy) and median total dose of 54 Gy (range 40.5–66.0 Gy). In 26 treatments (68%), a simultaneous integrated boost (SIB) was included. Patients with Ewing’s sarcoma (*n* = 23) received RT with a median total dose of 54 Gy (range 18–60 Gy) and a median single dose of 2 Gy (range 1.5–2.27 Gy). All 6 patients diagnosed with rhabdomyosarcoma (alveolar and embryonal) were treated with a median total dose of 50 Gy (range 44–50 Gy) and a single dose of 2 Gy. Patients who were diagnosed with other soft tissue sarcomas (*n* = 7) received RT with a median total dose of 60 Gy (range 50–66) and a single dose of 2 Gy. In both patients with osteosarcoma, RT was applied adjuvantly with a median total dose of 55 Gy (range 50–60 Gy). More details about dose and irradiated localization for each entity are shown in supplementary file 2. The median number of fractions included 25 fractions (range 10–30 fractions) with a median irradiation time of 8.4 min (range 3.5–34.8 min). The median time from diagnosis to RT was 4 months (range 1–16 months). Tumors were localized in the thorax (*n* = 24 [63%]), abdomen (*n* = 21 [55%]), and on extremities (*n* = 17 [45%]). Seven patients (18%) had RT in the area of the head and neck. However, 20 patients (53%) received mtRT. The reason for RT was primary disease in 28 patients (74%). Ten patients (26%) were treated for recurrences, of whom 5 patients had local relapses and 5 patients a systemic relapsed disease. A single patient had previously received RT 4 years in advance, undergoing reirradiation for local recurrence of an Ewing’s sarcoma. The indication for RT was set for each patient individually depending on age, entity, anatomic location, stage of disease, and the possibility of surgical removal. Seven patients (18%) received neoadjuvant RT to improve the success of surgical removal. If indicated, RT was applied adjuvantly after surgery (*n* = 23 [61%]), because lesions were not resectable with clear margins and had microscopically residual disease. In 21% of patients (*n* = 8), definitive RT was performed because of a nonresectable tumor due to its site and possible mutilation. Depending on the entity, chemotherapy was performed either within the Euro-Ewing study protocols (*n* = 22) or according to the CWS (*Cooperative Weichteilsarkomstudie*) guidance (*n* = 13). Six patients received standardized chemotherapy according the Euro-Ewing study ’99 (R1 group [*n* = 3], R2 group [*n* = 2], R3 group [*n* = 1]), whereas 16 patients were treated according to the Euro-Ewing study 2008 protocol (R1 group [*n* = 2], R2 group [*n* = 6], R3 group [*n* = 4], no further details [*n* = 4]). One patient received previous treatment as a very-high-risk patient within the CWS study 2004, another 4 patients were included in the very-high-risk group of the CWS study 2002, and 7 others received treatment according to the CWS 2007 HR protocol. Two patients with osteosarcoma were included in the Euroamoss-1/Cross study, and 1 patient with a multifocal Ewing sarcoma was treated according to the AEWS0031 study protocol. The majority of patients (*n* = 36) received chemotherapy before RT. Additionally, 29 patients (76%) had a concomitant radiochemotherapy (RCT). After RT, 17 patients (45%) underwent stem cell transplantation. Most patients (*n* = 35) were treated with curative intent, while 3 patients (8%) with recurrences received palliative care.

### Follow-up and toxicity

The occurring toxicity mentioned either during the radiotherapy session or within the follow-up period was protocolled prospectively in our institutional database or the patient’s chart. The first follow-up was scheduled 6–8 weeks after RT and included a contrast-enhanced CT in addition to the clinical assessment. During the first year, regular assessments were performed every 3 months, then every 6‑12 months depending on the clinical need of the patient and the prognosis. Toxicity during and after RT was graded according to the Common Terminology Criteria for Adverse Events (CTCAE) version 4.03 and classified into symptoms during RT, acutely (0–6 months) and late (>6 months) after RT, and long-term side effects (>24 months after RT). Patients were asked to estimate their quality of life (QoL) on a scale from 1 to 7 according to the European Organization for Research and Treatment of Cancer (EORTC) quality of life questionnaires (QLQ C30) version 3.0, with 1 indicating worst and 7 best QoL at the follow-up examination. The patients’ general health status and independence were measured according to the Karnofsky Performance Status Scale (KPS). Table 2 presents further patient characteristics.

**Table 2:** Patient characteristics

Characteristics			*n* (%)
Gender	Male	–	23 (60.5%)
	Female		15 (39.5%)
Age	Median age	–	15 (16–19) years
Tumor site /histology	*Head and neck*	–	*7 (18%)*
		Ewing’s sarcoma	2 (29%)
		Rhabdomyosarcoma	3 (43%)
		Desmoid sarcoma	1 (14%)
		Unclassified sarcoma	1 (14%)
	*Thorax*	–	*24 (63%)*
		Ewing’s sarcoma	17 (71%)
		Rhabdomyosarcoma	5 (21%)
		Osteosarcoma	1 (4%)
		Unclassified sarcoma	1 (4%)
	*Abdomen and pelvis*	–	*21 (55%)*
		Ewing’s sarcoma	13 (62%)
		Rhabdomyosarcoma	5 (24%)
		Osteosarcoma	2 (8%)
		Unclassified sarcoma	1 (4%)
	*Extremities*	–	*17 (45%)*
		Ewing’s sarcoma	10 (59%)
		Rhabdomyosarcoma	3 (18%)
		Synovial sarcoma	2 (12%)
		Fibromyxoid sarcoma	1 (6%)
		Desmoid sarcoma	1 (6%)
Treatment intention	Primary disease	–	28 (74%)
	Recurrent disease		10 (26%)
Chemotherapy	Before RT	–	36 (95%)
	Concomitant RCT		29 (76%)
Radiation treatment	Median single dose	–	2 Gy (1.80–2.27 Gy)
	Median total dose		54 Gy (40.5–66.0 Gy)
	Simultaneous boost		26 (68%)
	Sequential boost		5 (13%)
	Neoadjuvant RT		6 (16%)
	Adjuvant RT		23 (61%)
	Definitive RT		8 (21%)
	Stem cell transplantation therapy		17 (45%)

*RT* radiotherapy, *RCT* radiochemotherapy

### Statistical analysis

All data analyses were performed using SPSS statistical software (version 24.0; IBM Corp., Armonk, NY, USA). The Kaplan–Meier method provided estimates of overall survival (OS) and progression-free survival (PFS), and the log-rank test was used for comparison of survival curves. We performed univariate analysis to test the association between several clinical and treatment characteristics and ≥grade 3 toxicity. We used the non-parametric Mann–Whitney test for quantitative variables and Fisher’s exact test for qualitative variables. *P*-values ≤0.05 were considered to be statistically significant.

## Results

**Fig. 1:**
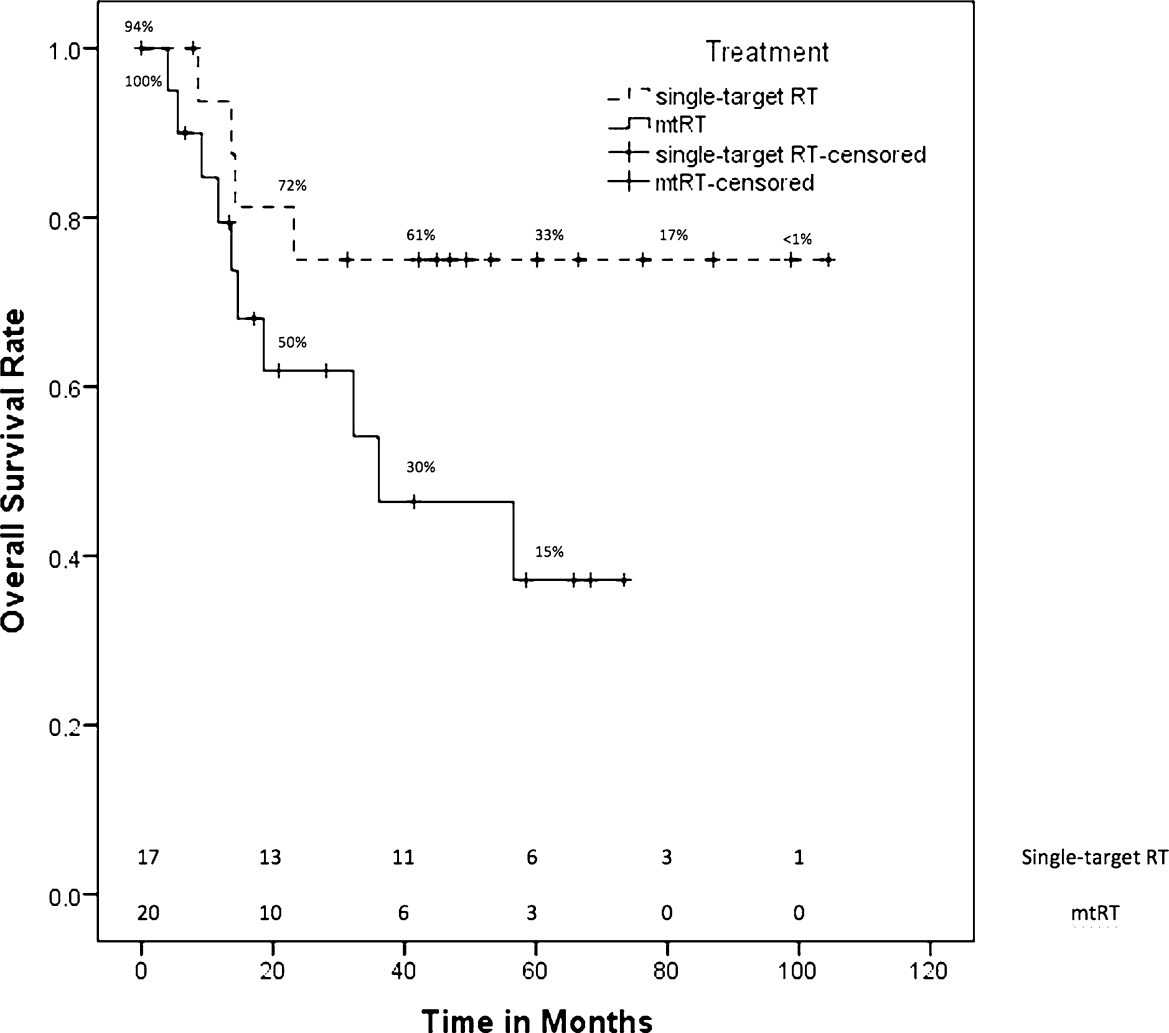
Kaplan–Meier estimator of overall survival of patients treated with multitarget radiotherapy (mtRT) and patients receiving single-target radiotherapy (RT)

**Fig. 2:**
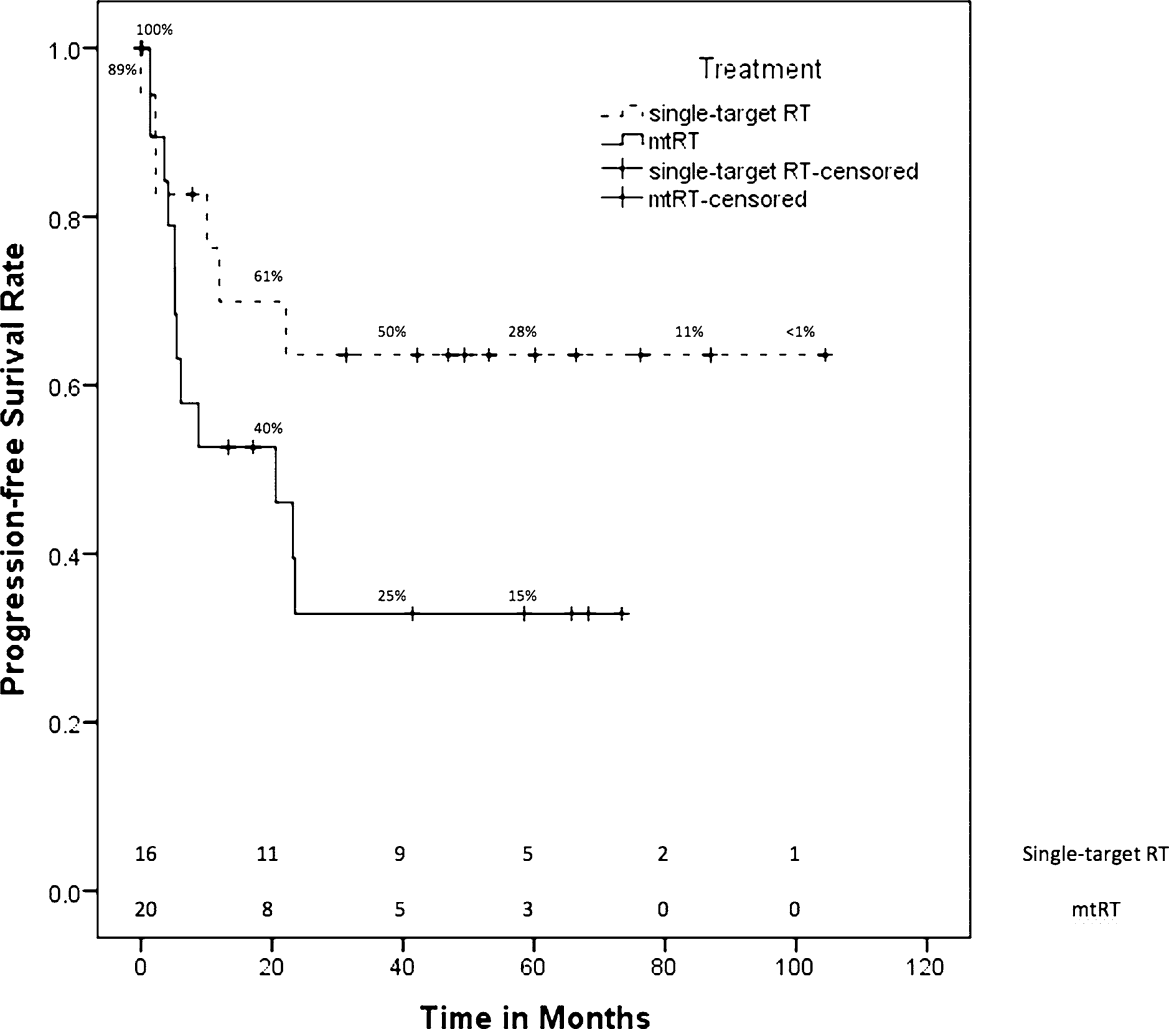
Kaplan–Meier estimator of progression-free survival of patients treated with multitarget radiotherapy (mtRT) and patients receiving single-target radiotherapy (RT)

The median follow-up after RT of all patients was 29.7 months (95% confidence interval [CI] 15.3–48.2 months). Supplementary file 1 lists the follow-up differentiated for each entity and RT group (single-target RT or mtRT). The 5‑year survival rate of all patients was 55.2 ± 9.5% and the mean PFS was 54.9 months (95% CI 38.9–71.1 months [median was not reached]). Fig. 1 displays overall survival rates for patients receiving mtRT and single-target RT. The 5‑year survival rate of all patients with mtRT (*n* = 20) was 37.1 ± 13.2%, while patients who received single-target RT (*n* = 18) had a 5-year survival rate of 75 ± 10.8%. Fig. 2 shows the PFS of both groups. Patients with single-target RT had a mean PFS of 69.5 months (95% CI 47.0–92.0 months [median was not reached]). The median PFS of all patients treated with mtRT was 20.6 months (95% CI 0.0–42.1 months). Nevertheless, there was no significant difference in OS (*p* = 0.076) or PFS (*p* = 0.111) due to high censorships. We evaluated 14 patients for side effects more than 2 years after RT. The median follow-up of the evaluable patients for long-term toxicities was 59.4 months (range 28.1–104.5 months).

### Multitarget radiation therapy

**Fig. 3:**
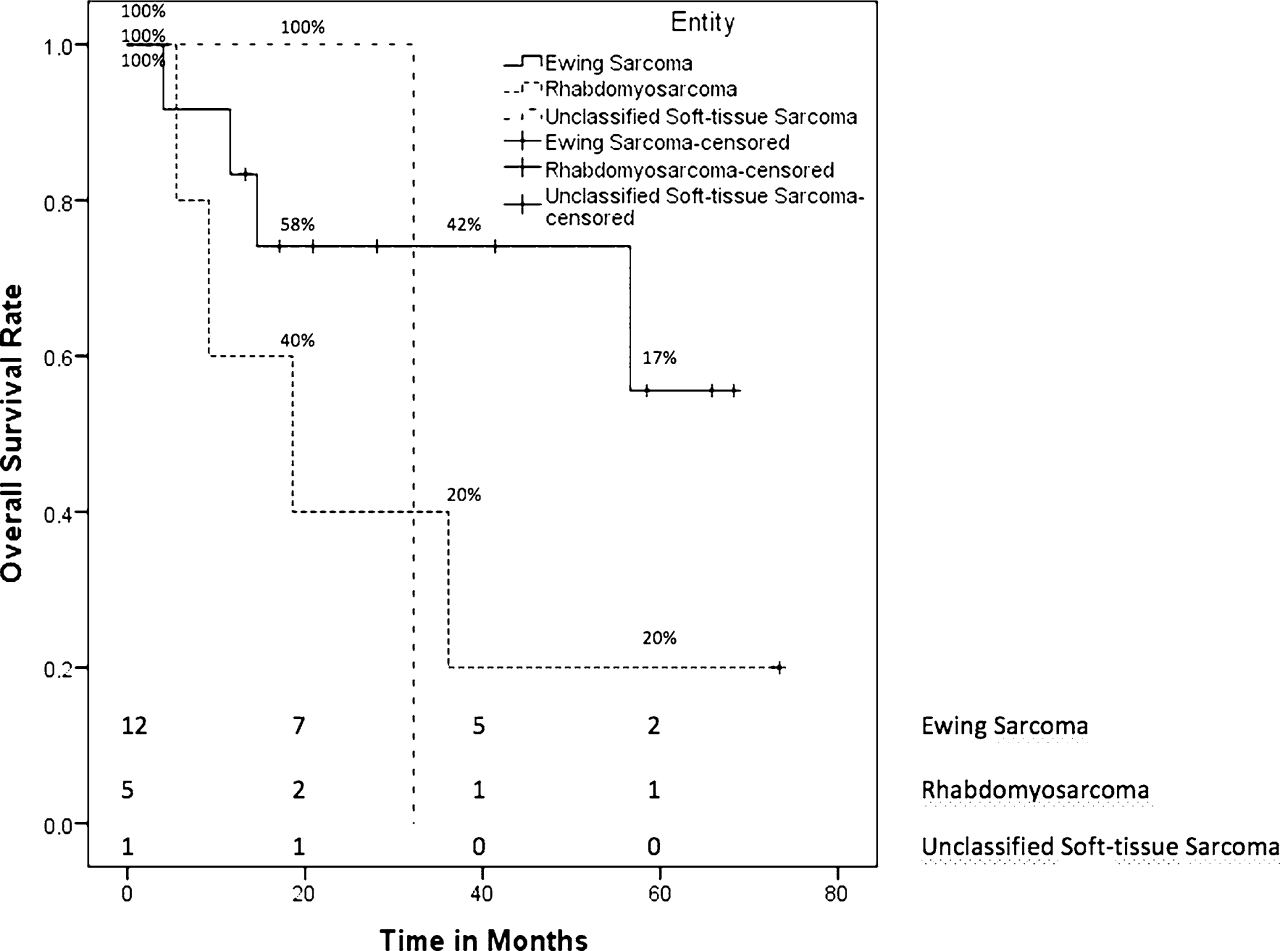
Kaplan–Meier estimator of overall survival of patients treated with mtRT for primary disease differentiated by entity

**Fig. 4:**
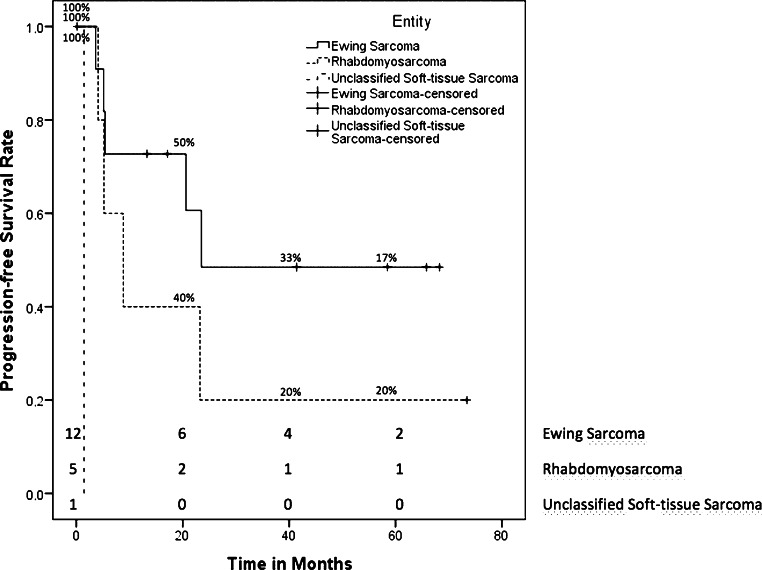
Kaplan–Meier estimator of progression-free survival of patients treated with mtRT for primary disease differentiated by entity

We treated 20 patients with mtRT and curative intent in our department. All patients had stage IV disease. Most patients (*n* = 18 [90%]) received mtRT for primary disease, but 2 patients (10%) for multifocal systemic relapses. The prevalent types of sarcomas were Ewing sarcoma (*n* = 14), four alveolar, and one embryonal rhabdomyosarcoma, as well as one unclassified sarcoma. Both patients with relapsed disease had a diagnosis of Ewing sarcoma. The median time from the date of diagnosis to RT was 4 months (range 3–16 months). The median OS of all mtRT patients was 36.1 months (95% CI 0.0–74.6 months). Fig. 3 shows the OS rates for patients receiving mtRT for primary disease differentiated by each entity. Patients diagnosed with Ewing sarcoma and mtRT for primary disease (*n* = 12) had a 5-year OS rate of 55.6% ± 18.7%. The OS of a patient with a relapsed Ewing sarcoma was 13.7 months; the other patient was censored due to loss to follow-up. The total Ewing sarcoma patients treated with mtRT had a 5-year OS rate of 51.1% ± 17.8%. Patients diagnosed with the primary disease of rhabdomyosarcoma (*n* = 5) had a 5-year OS rate of 20% ± 17.9%. The 1 patient with an unclassified soft tissue sarcoma showed an OS of 32.2 months. All patients received initial chemotherapy, but 19 patients (95%) had concomitant RCT. RT was performed with a median dose of 54 Gy (range 40.5–60.0 Gy) and a median single dose of 2 Gy (range 1.80–2.27 Gy). For more details on dose and localization, see supplementary file 2. The median follow-up after RT was 26.4 months (range 0.1–73.4 months). PFS differentiated by the entity for patients with a primary disease is displayed in Fig. 4. Patients with the primary disease of an Ewing sarcoma (*n* = 12) had a mean PFS of 39.7 months (95 CI 21.7–57.6 months), of whom 5 patients showed tumor progression (42%). Additionally, the PFS of patients with relapsed disease and Ewing sarcoma was 1.4 months and 6 months, respectively. Four patients (80%) with rhabdomyosarcoma had tumor progression after RT with a mean PFS of 22.9 months (95 CI 0–45.8 months). One patient with an unclassified soft tissue sarcoma had a PFS of 1.4 months.

The most common grade 1 and 2 toxicities during RT were nausea and vomiting (*n* = 12 [60%]), radiodermatitis (*n* = 11 [55%]), and fatigue (*n* = 9 [45%]). Nine patients (45%) showed hyperpigmentation of the skin and 9 patients (45%) had pain grade 1. Table 3 displays all severe non-hematological toxicities (grade ≥3) that occurred during and acutely after RT. Two patients’ (10%) KPS worsened to ≤40% during RT, with a significant relationship (*p* = 0.044) to the duration of RT in minutes. A low KPS was strongly associated with irradiation of tumors in the area of the head and neck (*p* = 0.053). Furthermore, the irradiated area of the head and neck had a strong association with severe mucositis (*p* = 0.053). Additionally, there was a significant correlation between the duration of RT in minutes and severe mucositis during RT (*p* = 0.044). Mucositis grade 4 of a patient receiving RT of the head and neck among other regions was the only non-hematological grade 4 toxicity that occurred during RT.

**Table 3:** Severe toxicity during and acutely after multitarget radiotherapy (mtRT)

	During RT (*n* = 20)		Acutely after RT (*n* = 18)	
Toxicity	Grade 3 Absolute (%)	Grade 4 Absolute (%)	Grade 3 Absolute (%)	Grade 4 Absolute (%)
Nausea/vomiting	2 (10%)	–	–	–
Loss of appetite	1 (5%)	–	–	–
Radiodermatitis	3 (15%)	–	2 (11%)	–
Mucositis	1 (5%)	1 (5%)	–	–
Dysphagia	2 (10%)	–	–	–
Diarrhea	1 (5%)	–	–	–
Urogenital infection	1 (5%)	–	–	–
Pain	2 (10%)	–	–	–
Hematological	10 (50%)	8 (40%)	–	–
Pericarditis	–	–	–	1 (6%)
Pericardial effusion	–	–	–	1 (6%)
Pneumonitis	–	–	1 (6%)	–
Enterocolitis	–	–	1 (6%)	–
Gastritis	–	–	1 (6%)	1 (6%)

Most patients (*n* = 18 [90%]) developed severe hematological toxicities during mtRT but with no significant relation to RT characteristics, see supplementary file 3. Further analysis showed that mtRT correlated highly significantly with severe thrombocytopenia during RT (*p* = 0.007). In 7 patients (35%), hematological toxicities led to an interruption of the RT procedure, but with no significant correlation (leukocytopenia [*p* = 1.000], thrombocytopenia [*p* = 0.650]). Those individual decisions were made upon close consultation with all professionals involved and responsible, and only if further irradiation could not be justified due to the unstable general condition. We evaluated 18 patients for acute toxicities after RT, with eight cases (44%) of severe side effects located in the abdominal region or thorax. None of those severe toxicities had a significant correlation with RT characteristics (see supplementary file 3). One patient with an unclassified sarcoma developed a grade 4 gastritis, pericarditis with pericardial effusion, but also an enterocolitis grade 3 within 6 months after RT. Another acute grade 3 gastritis occurred in a patient with alveolar rhabdomyosarcoma. Two patients with Ewing sarcomas in the area of the trunk suffered from grade 3 radiodermatitis acute after RT. One patient with a pulmonary spread Ewing sarcoma had a grade 3 pneumonitis within 6 months after RT. Two grade 3 side effects, one dyspnea and one pericardial effusion, occurred as late toxicity. Five patients were evaluated for long-term toxicities after mtRT, with a median follow-up of 65.8 months (range 28.1–73.4 months). Two patients (40%) had mild grade 1 dyspnea and 1 patient had a permanent grade 2 alopecia of the scalp. None of the patients indicated impairments (<6/7) of QoL or their health status for more than 2 years after mtRT.

### Single-target radiation therapy

**Fig. 5:**
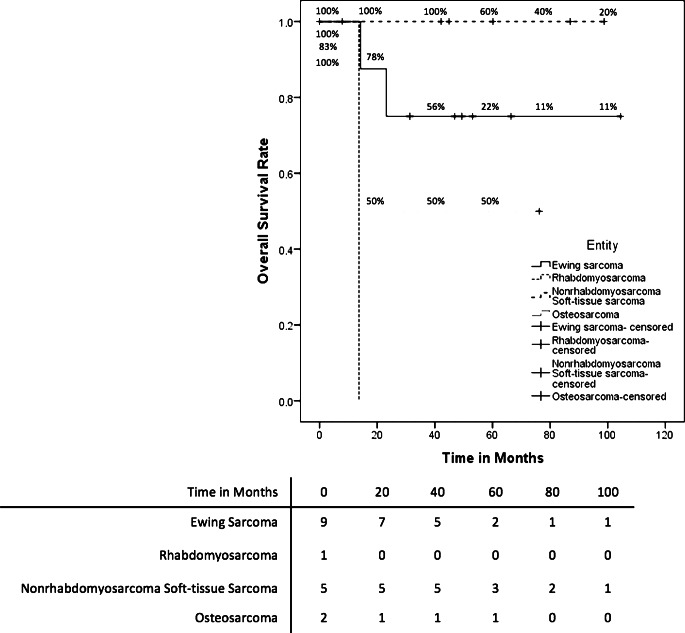
Kaplan–Meier estimator of overall survival of patients treated with single-target RT differentiated by entity

**Fig. 6:**
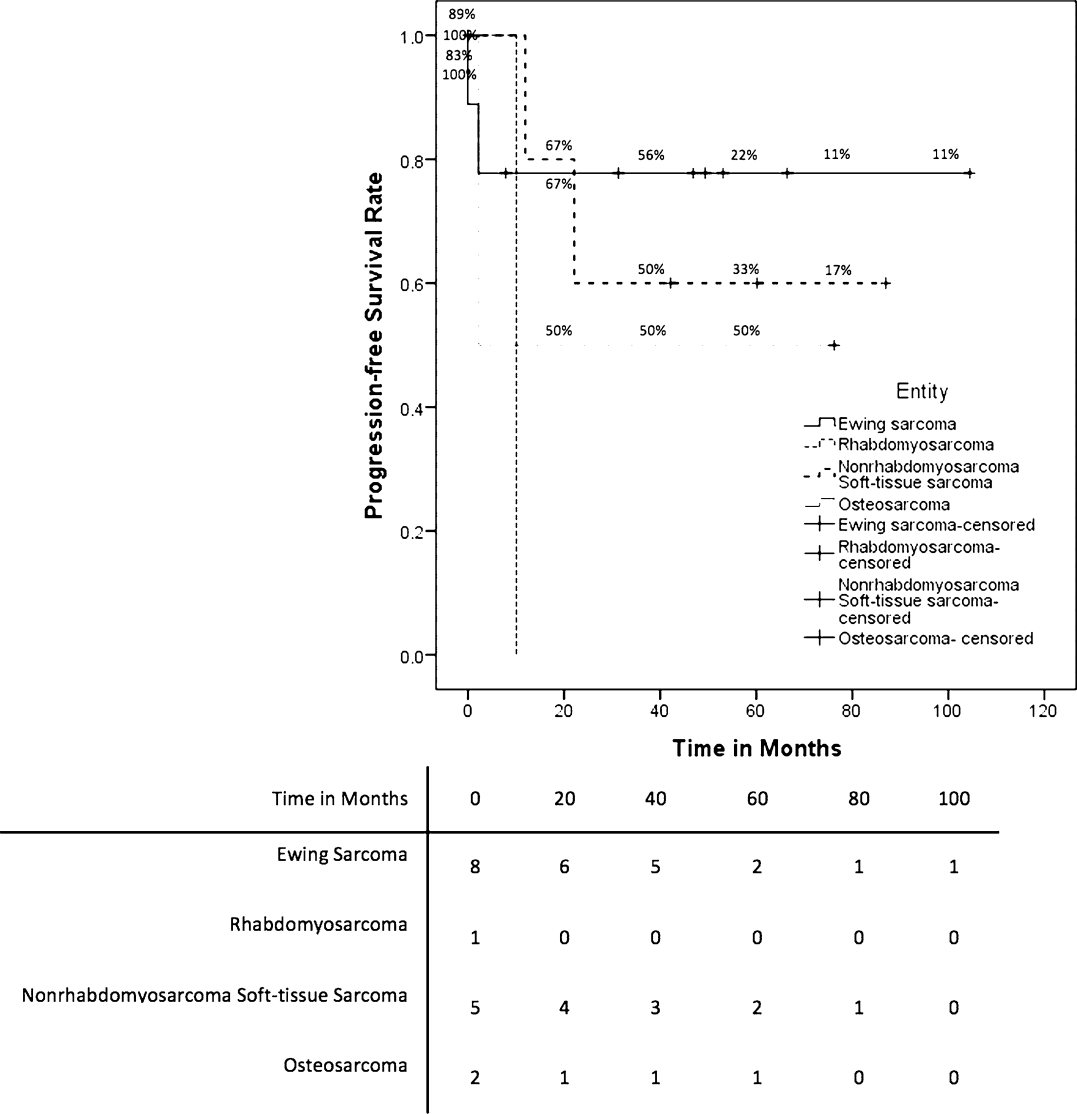
Kaplan–Meier estimator of progression-free survival of patients treated with single-target RT differentiated by entity

Most patients with single-target RT (*n* = 8 [44%]) had stage IV disease at diagnosis, 2 patients (11%) were in stage III, and 5 patients (28%) in stage II. Treatment intent was palliative in 3 patients (17%), all others (*n* = 15 [83%]) were treated curatively. The prevalent types of sarcomas were Ewing sarcoma (*n* = 9), osteosarcoma (*n* = 2), alveolar rhabdomyosarcoma (*n* = 1), and six other soft tissue sarcomas (desmoid sarcoma [*n* = 2], synovial sarcoma [*n* = 2], fibromyxoid sarcoma [*n* = 1], unclassified sarcoma [*n* = 1]). In 56% (*n* = 10), RT was performed for primary disease. Both osteosarcoma patients, 3 patients with Ewing sarcoma, and 3 patients with other soft tissue sarcomas (desmoid sarcoma [*n* = 2], fibromyxoid sarcoma [*n* = 1]) were treated for recurrences (*n* = 8 [44%]). All of the recurrences were local relapses, but 3 patients had RT for systemic relapsed metastasis: 1 patient received RT for a vertebral metastasis of osteosarcoma and 2 patients with Ewing sarcomas had RT for each lung metastasis or an osseous metastasis in the iliac bone. The median total dose was 54 Gy (range 45–66 Gy) with a median single dose of 2 Gy (range 1.8–2.1 Gy). The median time from the date of diagnosis to RT was 4 months (range 1–15 months) with a median follow-up after RT of 29.7 months (range 0–104.5 months). All patients receiving single-target RT had a mean OS of 82.1 months (95% CI 63.1–101.2 months [median was not reached]). The OS rates depending on entity are shown in Fig. 5. Patients diagnosed with Ewing’s sarcoma (*n* = 9) had a 5-year survival rate of 75% ± 15.3%. Six patients with Ewing sarcoma received single-target RT for primary disease, one of whom died after 23.2 months. One patient with recurrent disease of an Ewing sarcoma had an OS of 14.2 months. The OS of 1 patient with the primary disease of alveolar rhabdomyosarcoma was 13.7 months. All patients with nonrhabdomyosarcoma soft tissue sarcoma were still alive. The two patients diagnosed with recurrences of osteosarcoma had a mean survival of 42 months (95 CI 0–89.3 months). Almost all patients (*n* = 17 [94%]) received initial chemotherapy. Ten patients (56%) had concomitant RCT with a significant relationship to severe leukocytopenia during RT (*p* = 0.008). The PFS rates of all single-target RT patients depending on entity are shown in Fig. 6. The mean PFS of patients diagnosed with Ewing sarcoma and treated with single-target RT (*n* = 9) was 81.5 months (95 CI 53.4–109.6 months). Distinguished by primary and recurrent disease, Ewing sarcoma patients with primary disease (*n* = 6) had a mean PFS of 55.4 months (95 CI 35.5–75.2 months), with 1 patient having a local failure (17%). Patients with recurrent disease of Ewing sarcoma (*n* = 3) had a mean PFS of 70.3 months (95 CI 15.7–124.9 months), also with 1 patient with local failure (33%). The 1 patient with the primary disease of alveolar rhabdomyosarcoma showed a PFS of 101 months. All patients with soft tissue sarcomas had a mean PFS of 49.4 months (95 CI 18.9–79.8 months), but 2 patients showed tumor progression. Patients with a primary disease (*n* = 3) had a mean PFS of 65.3 months (95 CI 30.7–99.9 months) and patients with recurrent disease had a mean PFS of 27 months (95 CI 6–48 months). The mean PFS of the two osteosarcoma patients was 39.3 months (95 CI 0–90.5 months), with 1 patient having tumor progression after 2.1 months.

The most common grade 1 and 2 toxicities during RT were fatigue (*n* = 13 [72%]), radiodermatitis (*n* = 10 [56%]), and nausea and vomiting (*n* = 9 [50%]). Ten patients (56%) showed hypopigmentation of skin and 11 patients (61%) had pain grade 1 (*n* = 9) and 2 (*n* = 2) during RT. All patients had a KPS of ≥70% during RT. Table 4 displays all severe non-hematological toxicities that occurred during and acutely after RT. None of these severe side effects had a significant relationship to RT characteristics (supplementary file 4). One patient (6%) needed an interruption of the RT procedure due to severe pain in the abdominal region. No severe side effects occurred as late toxicity in single-target RT patients.

**Table 4:** Severe toxicity during and acutely after single-target radiotherapy (RT)

	During RT (*n* = 18)		Acute after RT (*n* = 15)	
Toxicity	Grade 3 Absolute (%)	Grade 4 Absolute (%)	Grade 3 Absolute (%)	Grade 4 Absolute (%)
Radiodermatitis	4 (22%)	1 (6%)	2 (13%)	–
Dysphagia	1 (6%)	–	–	–
Pain	1 (6%)	–	–	–
Hematological	8 (44%)	4 (22%)	–	–
Mucositis	–	–	1 (7%)	–
Loss of appetite	–	–	1 (7%)	–

Twelve patients (67%) developed hematological toxicities during RT, of whom 2 patients (11%) needed an interruption of the RT procedure due to strong leukocytopenia (*p* = 0.529). Severe leukocytopenia was not associated significantly with the applied total dose (*p* = 0.580) or RT duration in minutes (*p* = 0.467).

Nine patients were evaluated regarding long-term toxicities, of whom 3 patients (33%) noted mild grade 1 dyspnea. One patient (11%) mentioned a reduced QoL (3/7) and health status (4/7). Two patients (22%) showed growth retardation (<3 percentile under the growth curve). Both were diagnosed with Ewing’s sarcoma and received RT in the abdominal and pelvic region with a median dose of 55 Gy (range 54–56 Gy) and a single dose of 2 Gy. The median follow-up of the patients evaluable for long-term toxicities was 53.1 months (range 31.3–104.5 months).

## Discussion

Several studies have shown that IMRT provides not only effective treatment regarding the outcome but also a quality of life advantage by reducing the risk of side effects [18–20]. With the integration of IGRT and a helical delivery pattern, even more accurate RT of anatomically complex tumors is possible [21].

So far, a few studies have demonstrated the results of mtRT in adult cancer patients [22–24]. However, to our knowledge, this is the first study to present the outcome and toxicity rates of tomotherapy in pediatric patients with a focus on mtRT of sarcomas. Fogliata et al. have already compared different radiation techniques for selected pediatric patients and stated helical tomotherapy to be a satisfactory treatment method for pediatric patients with large complex-shaped tumors near organs at risk [25]. In this study, we analyzed 38 patients diagnosed with histologically different types of sarcomas. At the time of RT, most patients had stage III or IV disease (*n* = 30 [79%]), which is generally associated with poor outcome [26–28]. Hence, risking a highly toxic multimodal treatment with RT in a semi-curative intent was the only chance of cure in most cases. In our patient cohort, 20 patients received mtRT and 18 patients single-target RT. Since various entities located in different areas of the body were included in this study, it is unfeasible to compare the general OS and PFS of this study with the survival data of other studies. However, the 5‑year survival rate of patients with stage IV disease receiving mtRT was 37.1% ± 13.2% and had to be emphasized. More precisely, this study shows an excellent 5‑year survival rate of 55.6% ± 18.7% for patients with primary disease multifocal Ewing sarcoma receiving mtRT, especially when compared to the 3‑year OS of only 34% ± 4% for multifocal Ewing sarcoma demonstrated by Ladenstein et al. and the 3‑year OS of 40% shown by Pape et al. [27, 29]. Further, Hamilton et al. analyzed the outcome of pediatric Ewing sarcomas and stated a 5-year OS of 27% for patients with metastatic disease and 85% for localized disease, where again our abovementioned outcome for multifocal Ewing sarcoma has to be highlighted [30]. Regarding localized disease, patients receiving single-target RT had a noteworthy 5‑year OS rate of 75 ± 10.8% and mostly included Ewing sarcoma (*n* = 9) and nonrhabdomyosarcoma soft tissue sarcoma (*n* = 6). The outcome is moreover acceptable, since Spunt et al. and Williams et al. published a 5-year OS of 50–90% depending on the risk profile for localized nonrhabdomyosarcoma soft tissue sarcomas [2, 31]. However, due to small numbers of patients and the included different entities as well as primary and recurrent diseases, a comparison to survival rates of other studies might not be conclusive. Despite this, the mean OS of 3 patients with a relapsed disease of Ewing sarcoma of 59.3 months (95 CI 0–121.9 months) after single-target RT seems noticeably auspicious considering the poor 5‑year OS of <15% shown by Bacci et al. and Leavey at al.[32, 33]. Patients with metastatic rhabdomyosarcoma had a 5-year OS of 20% ± 17.9% that is comparatively similar in context to Rudzinski et al., who indicated an approximate OS of 20–40%, and Breneman et al., who mentioned a 3-year OS of 39% (95% CI 30–48%) [34, 35]. Besides, a good outcome and little toxicity of IMRT in comparison to other RT techniques were demonstrated by Qui et al. in pediatric nasopharyngeal carcinoma [36]. Therefore, it would be interesting to compare outcomes in pediatric patients with a focus on mtRT using tomotherapy to other radiation techniques in further studies. Due to improving outcomes in pediatric cancer patients, reduction of side effects is more and more important. Especially the risk of second malignancies is a major concern in the treatment of childhood cancers. Particularly in the field of radiation oncology, proton beam therapy offers another approach in the local treatment of pediatric cancers by irradiating sensitive tumor sites very precisely with only a low dose to surrounding healthy tissue and nearby organs at risk [37, 38]. Due to the excellent dose distribution in proton beam therapy, studies show promising results with low toxicity rates, but also indicate the need for further studies on the long-term toxicity of proton therapy in pediatric patients [39, 40]. Besides, proton therapy seems to mitigate the risk of developing second cancers as long-term toxicity after radiation compared to photon therapy [41]. In this study, however, the incidence of severe toxicity caused by tomotherapy was noticeably low, with no significant difference between both groups (single-target RT and mtRT). During RT, 13 patients (65%) with mtRT and 6 patients (33%) with single-target RT had grade 3 toxicity. However, especially non-hematological grade 4 toxicities occurred rarely (*n* = 2 [5%]) during RT. Such low rates of non-hematological grade 4 toxicity are even more surprising considering the poor prognosis of most patients with a need for aggressive RT (median dose 54 Gy) and concomitant chemotherapy (76%). Furthermore, the latter was the only significant factor associated with severe leucopenia during RT in patients receiving single-target RT. When comparing both subgroups concerning hematological toxicity, the percentage of patients receiving mtRT (*n* = 18 [90%]) was higher than that of patients with single-target RT (*n* = 12 [67%]). Lee et al. also mentioned high rates of severe leucopenia for patients undergoing mtRT [23]. In patients with mtRT, the duration of RT in minutes was significantly related to the KPS of the patient, due to the size of target lesions and, therefore, the severity of disease. After RT only a few patients presented acute severe toxicity. Grade 3 toxicity occurred in five cases acutely after mtRT and in four individual cases after single-target RT. Only 1 patient who had a multifocal unclassified soft tissue sarcoma suffered from several non-hematological grade 4 toxicities (gastritis, pericarditis, and pericardial effusion) acutely after mtRT. Patients with single-target RT had no acute non-hematological grade 4 toxicity. The same applies to severe toxicity as a late effect, with 1 patient suffering from severe grade 3 dyspnea after receiving mtRT for a multifocal and pulmonary spread Ewing sarcoma. Another patient had a grade 3 pericardial effusion as a late effect after mtRT for an alveolar rhabdomyosarcoma in the posterior mediastinum. Patients with single-target RT did not show any severe toxicity as a late effect.

This study is unique regarding the analysis of long-term sideeffects of tomotherapy. We evaluated 14 pediatric patients (5 patients with mtRT, 9 patients with single-target RT) more than 2 years after RT. No patient had severe long-term side effects of tomotherapy. However, 2 patients showed growth retardation (<3 percentile under the growth curve) after single-target RT for Ewing sarcomas in the abdominal and pelvic region, and 1 patient indicated restrictions in QoL and health status after receiving single-target RT for an Ewing sarcoma in the coccyx. In those individual cases, however, the severity of the highly advanced diseases with the need for aggressive and multimodal treatment should be taken into consideration. Remarkably, none of the 5 patients with multifocal tumor stages stated restrictions of their health status and their QoL according to EORTC QLQ C30 more than 2 years after mtRT. One major long-term toxicity in cancer treatment is the risk of second neoplasms. Especially children and adolescents are affected by the long-term side effects of RT and the dangers of developing second neoplasms and, therefore, mortality [42, 43]. We are aware that a median follow-up period of 29.7 months (95% CI 15.3–48.2 months) in this study only allows a limited statement on long-term toxicity and second neoplasms. Nevertheless, none of the 14 patients evaluable for long-term toxicities within a follow-up range up to 104.5 months (supplementary file 1) have developed second neoplasms after photon therapy so far. Moreover, severe late effects and long-term toxicities occurred only rarely.

We acknowledge that the limited patient number in our study leads to insufficient statistical power. Hence, further studies on long-term toxicities and outcomes of pediatric patients after tomotherapy and especially mtRT are needed in the future. In particular, concerning the risk of second malignancies after tomotherapy, a longer follow-up period is necessary for a relevant conclusion. It would also be helpful to analyze toxicities regarding different tumor sites as well as tumor entities, making a higher number of patients necessary.

In conclusion, our results show acceptable levels of acute and late toxicities considering the highly advanced diseases and multimodal treatment. Hence, tomotherapy is a suitable treatment method, especially for young patients with tumors of complex-shaped anatomy or multiple targets. Further analysis of long-term toxicity rates showed that side effects caused by RT are rare, despite the relatively short follow-up period. In particular, children with multifocal tumor stages treated with mtRT had no long-term side effects compared to patients receiving RT of just one tumor site. Furthermore, patients treated with mtRT showed a 5-year survival rate of 37.1 ± 13.2% and a median OS of 36.1 months (95% CI 0.0–74.6 months). Thus, mtRT is a promising approach and an innovative treatment method for pediatric sarcomas.

## Caption Electronic Supplementary Material

Supplementary file 1: Details on the median follow-up listed for each entity

Supplementary file 2: Radiotherapy characteristics for each entity

Supplementary file 3: *P*-values for severe toxicities (≥3 grade) in mtRT cases

Supplementary file 4: *P*-values for severe toxicities (≥3 grade) in single-target RT cases
