# Supplementary material for: Prospective evaluation of multitarget treatment of pediatric patients with helical intensity-modulated radiotherapy
Source: Strahlenther Onkol. 2020 Aug 3;196(12):1103–15. doi: 10.1007/s00066-020-01670-4 (PMC7686189; doi:10.1007/s00066-020-01670-4)
Supplement: Supplementary file 1 — Details on the median follow-up listed for each entity [file 66_2020_1670_MOESM1_ESM.docx]

Supplement File 1: Details on the median follow-up listed for each entity

| Entity | Median follow-up in total | Median follow-up after single-target RT | Median follow-up after mtRT |
| --- | --- | --- | --- |
| Ewing Sarcoma | n=23  28.1 months  (range, 0.1-104.5 months) | n=9  46.9 months  (range, 7.9-104.5 months) | n=14  19 months  (range, 0.1-68.3 months) |
| Rhabdomyosarcoma | n=6  14.2 months  (range, 5.2-73.4 months) | n=1  10.4 months | n=5  17.9 months  (range, 5.2-73.4 months) |
| Other Soft-tissue Sarcomas* | n=7  44.9 months  (range, 0-98.8 months) | n=6  52.6 months  (range, 0-98.8 months) | n=1  26.4 months |
| Osteosarcoma | n=2  41.8 months  (range, 7.4-76.3 months) | n=2  41.8 months  (range, 7.4-76.3 months) | - |
| * Fibromyxoid Sarcoma (n=1), Synovial Sarcoma (n=2), Desmoid Sarcoma (n=2), Unclassified Sarcoma (n=2) | | | |
