# Supplementary material for: Prospective evaluation of multitarget treatment of pediatric patients with helical intensity-modulated radiotherapy
Source: Strahlenther Onkol. 2020 Aug 3;196(12):1103–15. doi: 10.1007/s00066-020-01670-4 (PMC7686189; doi:10.1007/s00066-020-01670-4)
Supplement: Supplementary file 2 — Radiotherapy characteristics for each entity [file 66_2020_1670_MOESM2_ESM.docx]

Supplement File 2: Radiotherapy characteristics for each entity

| Ewing Sarcoma (n=23) | Area | Localization | | | Overall-dose in Gy (incl. Boost-Concept) | Single-dose in Gy |
| --- | --- | --- | --- | --- | --- | --- |
| Single-target RT (n=9) | Head and neck (n=1) | Cervical vertebral bodies | | | 54.0 | 2.0 |
|  | Extremities (n=2) | Lower extremities (n=1) | | | 45.0 | 2.0 |
|  |  | Upper extremities (n=1) | | | 54.0 | 2.0 |
|  | Thorax (n=2) | Osseous hemithorax (n=1) | | | 54.0 | 2.0 |
|  |  | Pleura incl. Mediastinum (n=1) | | | 60.0 | 2.0 |
|  | Pelvis (n=4) | Os ilium incl. joints (n=2) | | | 56.0 59.4 | 2.1 (range, 2.0-2.2) |
|  |  | Os coccygis (n=1) | | | 54.0 | 2.0 |
|  |  | Sacral vertebral bodies (n=1) | | | 45.0 | 1.8 |
| Multi-target RT (n=14) | Head and neck (n=1) | Calvaria | | | 39.6 | 1.8 |
|  | Extremities (n=8) | Lower extremities (n=6) | | | 56.7 (range, 46.0-60.0) | 2.0 (range, 1.8-2.0) |
|  |  | Upper extremities (n=2) | | | 45.0 | 1.9 (range, 1.8-2.0) |
|  | Thorax (n=14) | Lung metastasis (n=11) | | | 18.4 (range, 15.0-46.0) | 1.5 (range, 0.74-2.08) |
|  |  | Osseous Hemithorax incl. Pleura (n=3) | | | 50.0 (range, 40.5-54.0) | 2.0 (range, 1.8-2.27) |
|  |  | Thoracic vertebral bodies (n=3) | | | 50.0 (range, 46.0-50.0) | 2.0 |
|  | Abdomen/Pelvis (n=9) | Osseous pelvis (n=6) | | | 52.5 (range, 45.0-60.0) | 2.0 (range,1.8-2.2) |
|  |  | Lumbar vertebra bodies (n=2) | | | 48.0 (range, 46.0-50.0) | 2.0 |
|  |  | Soft tissue/Organs (n=2)  *(kidney, vessels, lymph nodes)* | | | 54.0 | 2.0 |
| Alveolar Rhabdomyosarcoma (n=5) | | |  |  |  |  |
| Single-target RT (n=1) | Thorax (n=1) | Pleura, diaphragm | | | 50.0 | 2.0 |
| Multi-target RT (n=4) | Head and Neck (n=3) | Lymph nodes, nasal/ maxillary sinuses | | | 44.0 (range, 44.0-50.0) | 2.0 |
|  | Extremities (n=2) | Upper extremities (n=2) | | | 44.0 | 2.0 |
|  | Thorax (n=3) | Lung metastasis (n=1) | | | 26.0 | 2.0 |
|  |  | Pleura incl. Mediastinum (n=2) | | | 47.0 (range, 44.0-50.0) | 2.0 |
|  |  | Thoracic vertebral bodies (n=1) | | | 44.0 | 2.0 |
|  | Abdomen/Pelvis (n=3) | Osseous pelvis (n=3) | | | 44.0 (range, 44.0-50.0) | 2.0 |
|  |  | Pancreatic head (n=1) | | | 44.0 | 2.0 |
| Embryonal Rhabdomyosarcoma (n=1) | |  | | | | |
| Multi-target RT (n=1) | Thorax (n=1) | Lung metastasis | | | 15.0 | 1.5 |
|  | Abdomen/Pelvis (n=1) | Bladder incl. lymph nodes | | | 50.0 | 2.0 |
|  |  | Os Ilium | | | 50.0 | 2.0 |
| Other Soft-tissue Sarcomas* (n=7) | |  | | |  |  |
| Single-target RT (n=6) | Head and Neck (n=1) | Nasal/ maxillary sinuses, orbit | | | 63.0 | 2.1 |
|  | Extremities (n=4) | Lower extremities (n=3) | | | 60.0 (range, 56.0-66.0) | 2.0 |
|  |  | Upper extremities (n=1) | | | 54.0 | 2.0 |
|  | Pelvis (n=1) | Os sacrum, lumbar vertebral bodies | | | 60.0 | 2.0 |
| Multi-target RT (n=1) | Head and Neck | Calvaria | | | 50.0 | 2.0 |
|  | Thorax | Mediastinum | | | 50.0 | 2.0 |
|  | Abdomen/Pelvis | Pancreatic head | | | 50.0 | 2.0 |
|  | Extremities | Lower extremities | | | 50.0 | 2.0 |
| Osteosarcoma (n=2) |  |  | | |  |  |
| Single-target RT (n=2) | Thorax (n=1) | Thoracic vertebral bodies | | | 60.0 | 2.0 |
|  | Abdomen/Pelvis (n=1) | Os sacrum | | | 50.0 | 2.0 |
| * Fibromyxoid Sarcoma (n=1), Synovial Sarcoma (n=2), Desmoid Sarcoma (n=2), Unclassified Sarcoma (n=2) | | | | | | |
