# Supplementary material for: Prospective evaluation of multitarget treatment of pediatric patients with helical intensity-modulated radiotherapy
Source: Strahlenther Onkol. 2020 Aug 3;196(12):1103–15. doi: 10.1007/s00066-020-01670-4 (PMC7686189; doi:10.1007/s00066-020-01670-4)
Supplement: Supplementary file 3 — P-values for severe toxicities (≥3 grade) in mtRT cases [file 66_2020_1670_MOESM3_ESM.docx]

Supplement File 3: P-values for severe toxicities (≥3 grade) in mtRT cases

| **Characteristics** | KPS after RT | Nausea and Vomiting | Radio-dermatitis | Leukocyto-penia | Thrombo- cytopenia | Dysphagia | Mucositis | Pain | Radio-dermatitis Acute | Gastritis Acute |
| --- | --- | --- | --- | --- | --- | --- | --- | --- | --- | --- |
| Age | 0.702 | 0.483 | 0.391 | 0.117 | 0.216 | 0.610 | 0.702 | 0.610 | 1.000 | 0.353 |
| Gender | 0.189 | 0.189 | 0.074 | 1.000 | 0.179 | 0.479 | 0.189 | 0.189 | 1.000 | 0.471 |
| Concomitant RCT | 1.000 | 1.000 | 1.000 | 1.000 | 1.000 | 1.000 | 1.000 | 1.000 | - | - |
| Stem cell transplantation after RT | 1.000 | 1.000 | 0.521 | 0.263 | 1.000 | 1.000 | 1.000 | 1.000 | 0.490 | 0.490 |
| RT: head and neck | 0.053 | 0.447 | 0.140 | 1.000 | 1.000 | 0.447 | 0.053 | 0.447 | 1.000 | 0.065 |
| RT: thorax | 0.100 | 1.000 | 0.150 | 1.000 | 0.474 | 1.000 | 0.100 | 1.000 | 1.000 | 1.000 |
| RT: abdomen | 1.000 | 0.521 | 1.000 | 0.316 | 0.350 | 0.521 | 1.000 | 0.521 | 1.000 | 0.529 |
| RT: extremities | 1.000 | 0.479 | 1.000 | 0.474 | 1.000 | 1.000 | 1.000 | 0.479 | 0.183 | 1.000 |
| RT duration (minutes) | 0.044 | 0.705 | 0.223 | 1.000 | 0.142 | 0.131 | 0.044 | 0.166 | 0.399 | 0.482 |
| Time between diagnosis and RT (months) | 0.144 | 0.550 | 0.265 | 0.332 | 0.794 | 0.550 | 0.144 | 0.144 | 0.375 | 0.554 |
| Total dose (Gy) | 0.249 | 0.249 | 0.282 | 0.926 | 0.647 | 0.249 | 0.249 | 0.109 | 0.156 | 0.256 |
| Interruption of RT procedure | 1.000 | 0.147 | 0.537 | 1.000 | 0.650 | 0.147 | 1.000 | 0.147 | 0.477 | 1.000 |
