# Supplementary material for: Prospective evaluation of multitarget treatment of pediatric patients with helical intensity-modulated radiotherapy
Source: Strahlenther Onkol. 2020 Aug 3;196(12):1103–15. doi: 10.1007/s00066-020-01670-4 (PMC7686189; doi:10.1007/s00066-020-01670-4)
Supplement: Supplementary file 4 — P-values for severe toxicities (≥3 grade) in single-target RT cases [file 66_2020_1670_MOESM4_ESM.docx]

Supplement File 4: P-values for severe toxicities (≥3 grade) in single-target RT cases

| Characteristics | Radio-dermatitis | Leukocytopenia | Thrombocytopenia | Dysphagia | Pain | Radio-dermatitis acute |
| --- | --- | --- | --- | --- | --- | --- |
| Age | 0.582 | 0.665 | 0.224 | 0.142 | 0.493 | 0.627 |
| Gender | 0.114 | 0.245 | 1.000 | 1.000 | 1.000 | 1.000 |
| Concomitant RCT | 0.608 | 0.008 | 1.000 | 1.000 | 1.000 | 1.000 |
| Stem cell transplantation after RT | 1.000 | 1.000 | 1.000 | 0.167 | 1.000 | 1.000 |
| RT: head and neck | 0.490 | 0.450 | 1.000 | 0.111 | 1.000 | 1.000 |
| RT: thorax | 0.249 | 0.516 | 1.000 | 1.000 | 1.000 | 1.000 |
| RT: abdomen | 0.314 | 1.000 | 1.000 | 1.000 | 0.444 | 0.467 |
| RT: extremities | 0.268 | 1.000 | 0.313 | 1.000 | 1.000 | 0.083 |
| RT duration (minutes) | 0.218 | 0.467 | 0.745 | 0.210 | 0.386 | 0.751 |
| Time between diagnosis and RT (months) | 0.081 | 0.579 | 0.271 | 0.845 | 0.242 | 0.809 |
| Total dose (Gy) | 0.653 | 0.580 | 0.124 | 0.625 | 0.282 | 0.873 |
| Interruption of RT procedure | 0.522 | 0.529 | 1.000 | 0.167 | 0.167 | 1.000 |
